# LDLR-related protein 10 (LRP10) regulates amyloid precursor protein (APP) trafficking and processing: evidence for a role in Alzheimer’s disease

**DOI:** 10.1186/1750-1326-7-31

**Published:** 2012-06-26

**Authors:** Julie Brodeur, Caroline Thériault, Mélissa Lessard-Beaudoin, Alexandre Marcil, Sophie Dahan, Christine Lavoie

**Affiliations:** 1 Department of Pharmacology, Faculty of Medicine and Health Sciences, Université de Sherbrooke, Sherbrooke, QC J1H 5 N4, Canada; 2 PerkinElmer, Montreal, QC, H3J 1R4, Canada

**Keywords:** LDLR-related protein 10 (LRP10), Amyloid precursor protein (APP), Amyloid beta (Aβ), Intracellular trafficking, Alzheimer’s disease, Endosome, Trans-Golgi network (TGN), Low density lipoprotein receptor (LDLR)

## Abstract

**Background:**

The Aβ peptide that accumulates in Alzheimer’s disease (AD) is derived from amyloid precursor protein (APP) following proteolysis by β- and γ-secretases. Substantial evidence indicates that alterations in APP trafficking within the secretory and endocytic pathways directly impact the interaction of APP with these secretases and subsequent Aβ production. Various members of the low-density lipoprotein receptor (LDLR) family have been reported to play a role in APP trafficking and processing and are important risk factors in AD. We recently characterized a distinct member of the LDLR family called LDLR-related protein 10 (LRP10) that shuttles between the trans-Golgi Network (TGN), plasma membrane (PM), and endosomes. Here we investigated whether LRP10 participates in APP intracellular trafficking and Aβ production.

**Results:**

In this report, we provide evidence that LRP10 is a functional APP receptor involved in APP trafficking and processing. LRP10 interacts directly with the ectodomain of APP and colocalizes with APP at the TGN. Increased expression of LRP10 in human neuroblastoma SH-SY5Y cells induces the accumulation of mature APP in the Golgi and reduces its presence at the cell surface and its processing into Aβ, while knockdown of LRP10 expression increases Aβ production. Mutations of key motifs responsible for the recycling of LRP10 to the TGN results in the aberrant redistribution of APP with LRP10 to early endosomes and a concomitant increase in APP β-cleavage into Aβ. Furthermore, expression of LRP10 is significantly lower in the post-mortem brain tissues of AD patients, supporting a possible role for LRP10 in AD.

**Conclusions:**

The present study identified LRP10 as a novel APP sorting receptor that protects APP from amyloidogenic processing, suggesting that a decrease in LRP10 function may contribute to the pathogenesis of Alzheimer’s disease.

## Introduction

Amyloid-β **(**Aβ) peptide accumulation in the brain is central to the pathogenesis of Alzheimer’s disease (AD). Aβ is produced by the serial proteolysis of amyloid precursor protein (APP) by secretases [1]. APP processing to amyloidogenic and non-amyloidogenic products is greatly affected by the subcellular localization of APP, presumably because of the specific subcellular localizations of secretases [2]. Non-amyloidogenic processing occurs mainly at the cell surface, where α-secretase and γ-secretase cleave APP into a soluble sAPPα fragment and non-toxic peptide p3. Amyloidogenic processing involves transit through endocytic organelles, where APP encounters β- and γ-secretases that cleave it into a soluble sAPPβ fragment and toxic Aβ peptides [2]. APP-interacting proteins that alter APP trafficking thus impact Aβ production.

Many members of the low-density lipoprotein receptor (LDLR) family interact with APP and regulate its intracellular trafficking [3]. The LDLR family consists of a large class of surface receptors usually involved in endocytosis and lipid metabolism [4]. However, recent reports have indicated that various members of this family play a role in APP trafficking and processing and are important risk factors in AD (reviewed in [2]). LDLR-related protein 1 (LRP1), a multifunctional endocytic receptor, interacts with APP and facilitates its internalization in endosomes and, consequently, its cleavage into Aβ [5]. Genetic studies have also linked LRP1 to AD [6]. SorLA (also called LR11 or SorL1) is a unique member of the LDLR family that is mainly localized in the trans-Golgi network (TGN) and endosomes [7]. SorLA interacts with APP and acts as a retention factor for APP in the Golgi, reducing its processing by secretases [8-10]. Evidence of decreased levels of SorLA in the brains of AD patients as well as epidemiological studies [8,11] point to an association between SorLA and AD.

Human LDLR-related protein 10 (LRP10, called LRP9 in mice) is a member of a new subfamily of LDLR that includes two other receptors, LRP3 and LRP12 [12]. This unique subfamily of LDLR is characterized by extracellular CUB domains and large cytoplasmic tails containing acidic dileucine (DXXLL) motifs [13]. Little is known about LRP10 apart from the fact that it is expressed in various tissues (including the brain), may be involved in apolipoprotein internalization [14], and is localized in the TGN and endosomes [13,15]. Two DXXLL motifs in the cytoplasmic tail of LRP10 interact with the clathrin adaptor GGA and AP proteins and are involved in LRP10 shuttling between the TGN and endosomes [13,15]. This strongly suggests that LRP10 plays a role in ligand trafficking between these intracellular compartments.

In the present study, we tested the hypothesis that LRP10 is a novel APP receptor involved in APP trafficking and processing to Aβ and explored its potential involvement in AD.

## Results

### LDLR-related protein 10 (LRP10) is a novel APP-interacting protein

**Figure 1:**
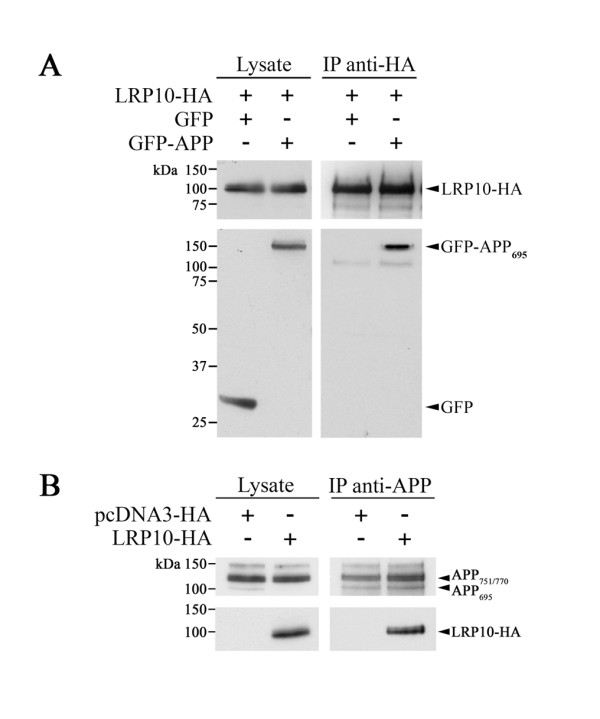
**LRP10 interacts with APP. (A)***In vivo* interaction of LRP10-HA and GFP-APP_695_ proteins. Lysates of HEK cells transfected with HA-tagged LRP10 and GFP or GFP-tagged APP were immunoprecipitated with anti-HA and then immunoblotted with anti-HA or anti-GFP antibody to detect LRP10 and GFP, respectively. **(B)** LRP10-HA interacts with endogenous APP. Lysates of HEK cells transfected with pcDNA3-HA or LRP10-HA were immunoprecipitated with anti-APP antibody and then immunoblotted with anti-HA antibody.

We initially evaluated the ability of LRP10 to bind APP since such binding would indicate that LRP10 plays a role as an intracellular APP receptor. We first performed immunoprecipitation experiments on HEK293 cells transfected with HA-tagged LRP10 and green fluorescent protein (GFP) or GFP-tagged APP. LRP10-HA coprecipitated with GFP-APP but not with GFP when we performed the immunoprecipitation with anti-HA immunoglobulin (IgG) (Figure 1A). This interaction was confirmed using untagged APP (Additional file 1: Figure S1) and using a combination of lysates from cells that expressed LRP10-HA and GFP-APP separately (Additional file 2: Figure S2). These results indicated that overexpressed APP and LRP10-HA interact within cell lysates. We next looked at whether endogenous APP interacts with LRP10 in HEK cells transfected only with HA-tagged LRP10. LRP10-HA coimmunoprecipitated with APP in the presence of anti-APP, confirming that LRP10 interacts specifically with endogenous APP (Figure 1B).

**Figure 2:**
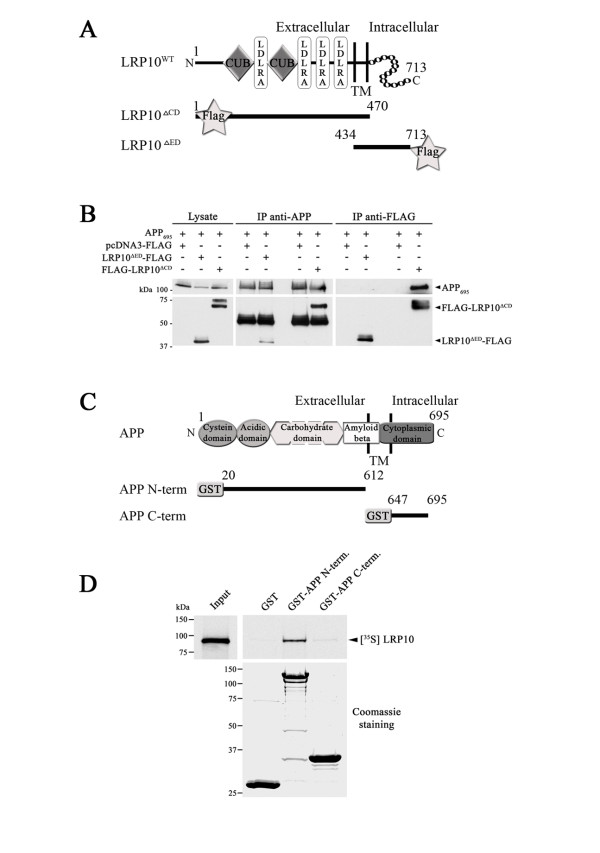
**Interaction of the ectodomains of LRP10 and APP. (A)** Schematic representation of the LRP10 FLAG-tagged deletion mutants used to determine the binding domain of APP. The structural elements of LRP10 are depicted, including (from amino to carboxyl terminus) the CUB domains, LDLRA repeat, and transmembrane (TM) domain. CD, Cytoplasmic domain; ED, Ectodomain. **(B)** The interaction between LRP10 and APP does not depend on the cytoplasmic domain of LRP10. Lysates of HEK cells transfected with APP_695_ and pcDNA3-FLAG, FLAG-tagged LRP10^ΔCD^, or FLAG-tagged LRP10^ΔED^ were immunoprecipitated with anti-APP or anti-FLAG and were immunoblotted with anti-APP or anti-FLAG antibody. **(C)** Schematic representation of the GST-APP deletion mutants used to determine the binding domain of LRP10. The structural elements of APP are depicted, including the cysteine, acidic, carbohydrate, amyloid beta, and cytoplasmic domains. **(D)***In vitro* interaction of LRP10 with the ectodomain of APP. The APP deletion mutants shown in (C) and the GST protein (10 μg each) were immobilized on glutathione beads and were incubated with *in vitro* translated ^35^ S-labeled LRP10. Bound proteins were separated by SDS-PAGE and were detected by autoradiography. GST proteins were detected by coomassie staining. Input equaled 2.5% of the total *in vitro*-translated product.

The association of LRP10 with APP may occur via contacts in the extracellular (luminal) and/or cytoplasmic regions of both proteins. To determine the importance of the extracellular and intracellular domains of LRP10 for the interaction with APP, we transfected HEK293 with APP_695_ together with FLAG-tagged LRP10 mutants that lacked either the cytoplasmic domain (LRP10^ΔCD^) or the extracellular or ectodomain (LRP10^ΔED^) (Figure 2A). Immunoprecipitations were performed with either anti-APP or anti-FLAG antibodies. A weak interaction was detected between APP_695_ and LRP10 ^ΔED^ while a stronger interaction was observed between APP_695_ and LRP10 ^ΔCD^ (Figure 2B), suggesting that the ectodomain of LRP10 is the major determinant for the interaction between LRP10 and APP. Lastly, we used pull-down assays to verify the *in vitro* interactions between LRP10 and the extracellular (luminal) region of APP (GST-APP N-term) and the cytoplasmic region of APP (GST-APP C-term) (Figure 2C). ^35^ S-labeled *in vitro*-translated LRP10 bound strongly to GST-APP-N-terminus and weakly to GST-APP-C-terminus compared to GST alone (Figure 2D). These results indicated that LRP10 interacts directly and predominantly with the ectodomain of APP *in vitro*.

### LRP10 colocalizes with APP in the TGN

**Figure 3:**
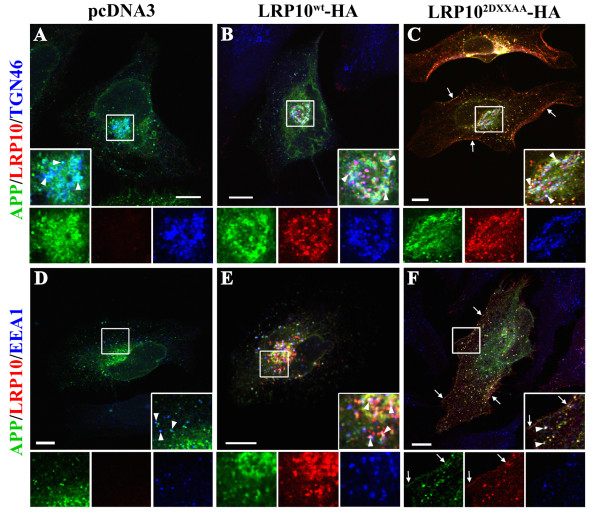
**LRP10 colocalizes with APP and modulates its intracellular distribution.** HeLa cells were transfected with GFP-APP and control vector pcDNA3 (A, D), wild-type LRP10-HA, (LRP10^wt^-HA; B, E), or HA-tagged-LRP10 in which two DXXLL motifs (that bind the clathrin adaptors GGAs and AP1/2) in the cytoplasmic tail were mutated (LRP10^2DXXAA^-HA, C, F). Cells were fixed, permeabilized, and immunostained with anti-GFP (green), anti-HA (red), and anti-TGN46 or EEA1 (blue) antibodies. The labeled cells were examined by confocal fluorescence microscopy. **(A, D)** In control pcDNA3 cells, APP-GFP was detected mainly in the juxtanuclear region and surrounding vesicles where it partially overlapped with TGN46 (A, inset, arrowheads) and EEA1 (D, inset, arrowheads). **(B, E)** LRP10^wt^-HA was detected in the juxtanuclear region and surrounding vesicles and partially overlapped with TGN46 (B) and EEA1 (E). In these cells, GFP-tagged APP was detected mainly in the Golgi region, where it partially overlapped with LRP10^wt^-HA (B). The merged images show a partial overlap between LRP10^wt^-HA and GFP-APP in the Golgi cisternae labeled by TGN46 (B, inset, arrowheads) and surrounding endosomes labeled by EEA1 (E, inset, arrowheads). **(C, F)** HA-tagged LRP10^2DXXAA^ was redistributed to the plasma membrane (arrows) and peripheral early endosomes (F, inset). In these cells, GFP-APP was also detected on the plasma membrane (F, inset, arrow), Golgi (C, inset) and in peripheral endosomes labeled by EEA1 (F, inset), where it colocalized with LRP10^2DXXAA^-HA (F, arrowheads, inset). Scale bar, 10 μm.

To determine whether LRP10 colocalizes with APP, we compared the intracellular distributions of HA-tagged LRP10 and GFP-tagged APP in HeLa cells by confocal microscopy. We mainly detected wild-type LRP10-HA in the juxtanuclear region and surrounding vesicles that codistribute with TGN46 and EEA1, which are markers of the trans-Golgi network (TGN) and early endosomes, respectively (Figure 3B, E). This was in agreement with our previous findings showing that LRP9, the mouse homolog of LRP10, localizes in the TGN and in endosomes in the Golgi area [13]. GFP-tagged APP was concentrated in the Golgi region, where it partially colocalized with LRP10^wt^-HA and TGN46 (Figure 3B, Merge) as well as surrounding EEA1-labeled vesicles (Figure 3E, Merge). The distribution of GFP-APP in cells expressing LRP10^wt^-HA was not mainly different from the distribution observed in control cells (Figure 3A, D). The results of the IF and biochemical interaction assays (immunoprecipitation and pull-down) suggested that LRP10 interacts with APP in the TGN cisternae and in the surrounding endosomes.

### LRP10 affects the intracellular distribution of APP in HeLa cells

To investigate the functional role of LRP10 on APP intracellular transport, we examined the subcellular distribution of APP in HeLa cells expressing an LRP10-trafficking mutant (LRP10^2DXXAA^). We and others recently showed that the substitution of leucines by alanines in two DXXLL motifs of the cytoplasmic tail of LRP9 (mouse homolog of LRP10) inhibits the interaction of LRP9 with clathrin adaptors AP-1/AP-2 and GGAs (Golgi-localized, γ-ear containing ARF-binding proteins) and causes its mislocalization to the endosomes and plasma membrane (PM) [13,15]. In concordance with the fact that the cytoplasmic tail of LRP10 has 83% homology with LRP9, the mutation of the two DXXLL motifs conserved in the cytoplasmic tail of LRP10 (LRP10^2DXXAA^) induced its redistribution to the cell surface and to peripheral punctate structures (Figure 3C, F). As previously observed with LRP9^2DXXAA^[13], a confocal microscopic assessment of the distribution of HA-tagged LRP10^2DXXAA^ with TGN46 and EEA1 confirmed that the DXXAA mutations shifted the distribution of LRP10 from the TGN to the PM and peripheral early endosomes (Figure 3F). Interestingly, the intracellular distribution of GFP-APP was modified in cells expressing the LRP10^2DXXAA^-trafficking mutant. In addition to its distribution in the Golgi region (Figure 3C), GFP-APP colocalized with LRP10^2DXXAA^ in peripheral EEA1-labeled endosomes and at the PM (Figure 3F). Furthermore, co-immunoprecipitation assays confirmed that HA-tagged LRP10^2DXXAA^ interacted with APP (Additional file 1: Figure S1). We concluded that the DXXLL motifs are required for the proper trafficking of LRP10 but not for the formation of the LRP10:APP complex. As such, the LRP10^2DXXAA^ mutant was able to redistribute APP to peripheral early endosomes.

**Figure 4:**
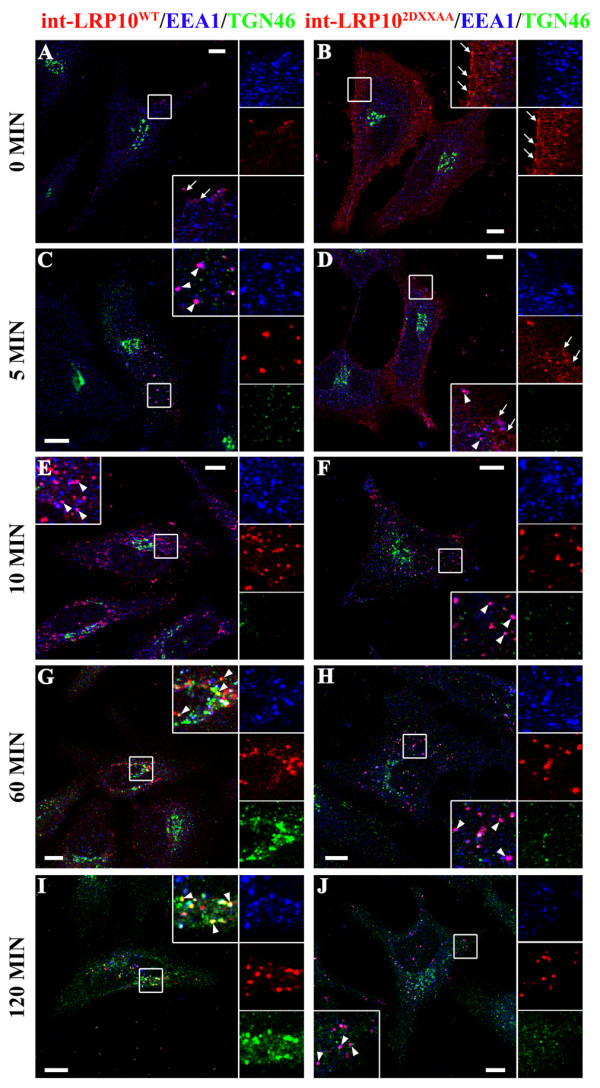
**Comparison of the intracellular trafficking pathways of LRP10**^**wt**^**and LRP10**^**2DXXAA**^**.** The internalization of LRP10 was evaluated in HeLa cells transfected with LRP10^wt^ or LRP10^2DXXAA^ tagged with an extracellular FLAG epitope. The cells were pre-incubated on ice to arrest endocytosis, and LRP10 molecules exposed on the cell surface were immunolabeled with antiserum directed against FLAG at 4°C. The cells were then washed and incubated at 37°C to allow internalization. Endocytosis and TGN targeting of cell surface-labeled LRP10 were evaluated after 0, 5, 10, 60, and 120-min chases at 37°C. The cells were then fixed, permeabilized, and immunostained with anti-TGN46 (green) or anti-EEA1 (blue) antibodies. The labeled cells were examined by confocal fluorescence microscopy. In cells expressing FLAG-LRP10^wt^, low level of FLAG-LRP10^wt^ was observed at the cell surface at time 0 (**A**, inset, arrows). Internalized LRP10^wt^ (int-LRP10^wt^) was observed in vesicles near the plasma membrane following a 5-min **(C)** and a 10-min **(E)** chase where it colocalized with EEA1 (**C**, **E**, insets, arrowheads). After a 60-min chase, int-LRP10^wt^ localized in the Golgi region and partially colocalized with the TGN marker TGN46 (**G**, inset, arrowheads). This colocalization was higher after a 120-min chase (**I**, inset, arrowhead). In cells expressing FLAG-LRP10^2DXXAA^, large amounts of LRP10^2DXXAA^ were detected on the cell surface at time 0 (**B**, arrows). After a 5-min chase, int-LRP10^2DXXAA^ was still distributed at the plasma membrane (**D**, arrow) or partially colocalized with EEA1 in vesicles near the PM (**D**, inset, arrowheads). After a 10-min chase, int-LRP10^2DXXAA^ was observed in EEA1-labeled vesicles (**F**, inset, arrowheads). However, after a 60-min chase **(H)**, and even a 120-min chase **(J)**, int-LRP10^2DXXAA^ was still mainly observed in early endosomes (**H**, **J**, insets, arrowheads). Scale bar, 10 μm.

To better understand how the LRP10^2DXXAA^ mutant affects the distribution/trafficking of APP, we first investigated the precise intracellular trafficking pathways of LRP10^wt^ and of LRP10^2DXXAA^ in order to precisely define the intracellular trafficking step impaired by the DXXLL motifs mutation. Since LRP10^wt^ is intracellular while the LRP10^2DXXAA^ mutant displays strong cell surface and endosome localization, it is possible that LRP10 constantly cycles between the TGN and the endosomes by transiting through the plasma membrane and that the mutation of both DXXLL motifs delays either its internalization and/or its retrograde transport from the endosome to the Golgi. Alternatively, it is possible that LRP10^wt^ never reaches the plasma membrane and that the DXXLL mutations promote the exit from the TGN and default transport to the plasma membrane. To distinguish between these two possibilities, we examined the presence of LRP10^wt^ at the cell surface of HeLa cells transfected with LRP10^wt^ tagged with an extracellular FLAG epitope. The cells were incubated with a specific anti-FLAG monoclonal antibody at 4°C to label the FLAG-LRP10^wt^ at the cell surface. The cells were then incubated at 37°C, and the uptake of cell-surface labeled FLAG-LRP10^wt^ was analyzed. At the initial time point, low level of FLAG-LRP10^wt^ was observed at the cell surface (Figure 4A). After 5 or 10-min chase, cell-surface labeled FLAG-LRP10^wt^ was observed in EEA1-labeled endosomes (Figure 4C, E). After a 60-min chase, it accumulated in the Golgi region, where it partially colocalized with TGN46 (Figure 4G), while after a 120 min chase, it clearly colocalized with TGN46 (Figure 4I). Large amounts of LRP10^2DXXAA^ tagged with an extracellular FLAG epitope were detected on the cell surface (Figure 4B). The uptake analysis of cell surface labeled FLAG-LRP10^2DXXAA^ showed that it was still mainly distributed at the plasma membrane after a 5-min chase (Figure 4D) but was clearly present in endosomes after a 10-min chase (Figure 4F), suggesting that its internalization was delayed compared to FLAG-LRP10^wt^. However, int-LRP10^2DXXAA^ was still present in this compartment after a 60 and 120-min chase (Figure 4H, J). This result suggested that the normal trafficking itinerary of LRP10 involves a transit to the plasma membrane, internalization, and retrograde transport from the endosome to the Golgi. The LRP10^2DXXAA^ trafficking mutant would also reach the PM and be internalized slowly into early endosomes. However, its exit from this compartment would be compromised. These results suggested that LRP10 moves through the secretory pathway to the cell surface. After rapid internalization in early endosomes, LRP10 is recycled back to the Golgi, a step that requires the DXXLL motifs in the cytoplasmic tail of LRP10 .

**Figure 5:**
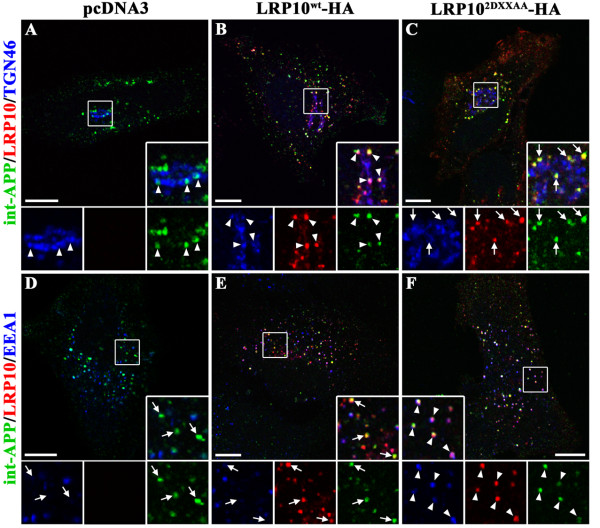
**LRP10**^**2DXXAA**^**inhibits the retrograde transport of APP from the endosome to the TGN.** Internalization of APP was evaluated in HeLa cells transfected with APP_695_ and pcDNA3 **(A, D)**, HA-tagged LRP10^wt^**(B, E)**, or LRP10^2DXXAA^**(C, F)**. The cells were pre-incubated on ice to arrest endocytosis, and APP molecules exposed on the cell surface were immunolabeled with antiserum directed against APP (α-22 C11) at 4°C. Endocytosis and TGN targeting of APP were evaluated after 60-min chase periods at 37°C. Cells were then fixed, permeabilized, and immunostained with anti-HA (red) and anti-TGN46 or anti-EEA1 (blue) antibodies. The labeled cells were examined by confocal fluorescence microscopy. In cells expressing pcDNA3 and LRP10^wt^-HA, internalized APP (int-APP, green) was localized in the Golgi region and partially colocalized with the TGN marker TGN46 (A, B, arrowheads). However, in cells expressing LRP10^2DXXAA^-HA, internalized APP (int-APP) was distributed mainly in vesicles labeled with the endosomal marker EEA1 and LRP10^2DXXAA^-HA (F, arrowheads). Arrowheads indicate structures in which APP colocalized with TGN46 or EEA1 while arrows indicate structures in which APP and TGN46 or EEA1 did not colocalize. Scale bar, 10 μm.

To determine whether LRP10 participates in the internalization and retrograde transport of cell surface APP to the TGN, we next analyzed the uptake of cell surface-labeled APP in cells overexpressing APP_695_ and HA-tagged LRP10^wt^ or LRP10^2DXXAA^ (Figure 5A-F). No altered internalization behavior was seen for APP coexpressed with LRP10^wt^ since cell surface labeled-APP colocalized with TGN46 after a 60-min incubation (Figure 5B, arrowheads), as observed in control cells (Figure 5A, arrowheads), indicating that APP reached the Golgi. However, in cells expressing LRP10^2DXXAA^, APP colocalized with EEA1 (Figure 5F, arrowheads) and did not redistribute to the TGN, even after 60 min (Figure 5C, arrows), indicating that APP was retained in the endosomes. We noticed that the half-life of APP is extended in this assay since cell-surface labeled APP could still be detected in the cells after 60 min internalization. This could be explained by the high amount of internalized APP due to overexpression or the presence of anti-APP antibodies internalized with APP that could affect or delay the processing of APP. In summary, these results suggested that LRP10 activity does not influence APP uptake from the cell surface while LRP10^2DXXAA^ inhibits the retrograde transport of APP from the endosome to the TGN.

### LRP10 affects the trafficking of endogenous APP in SH-SY5Y cells

**Figure 6:**
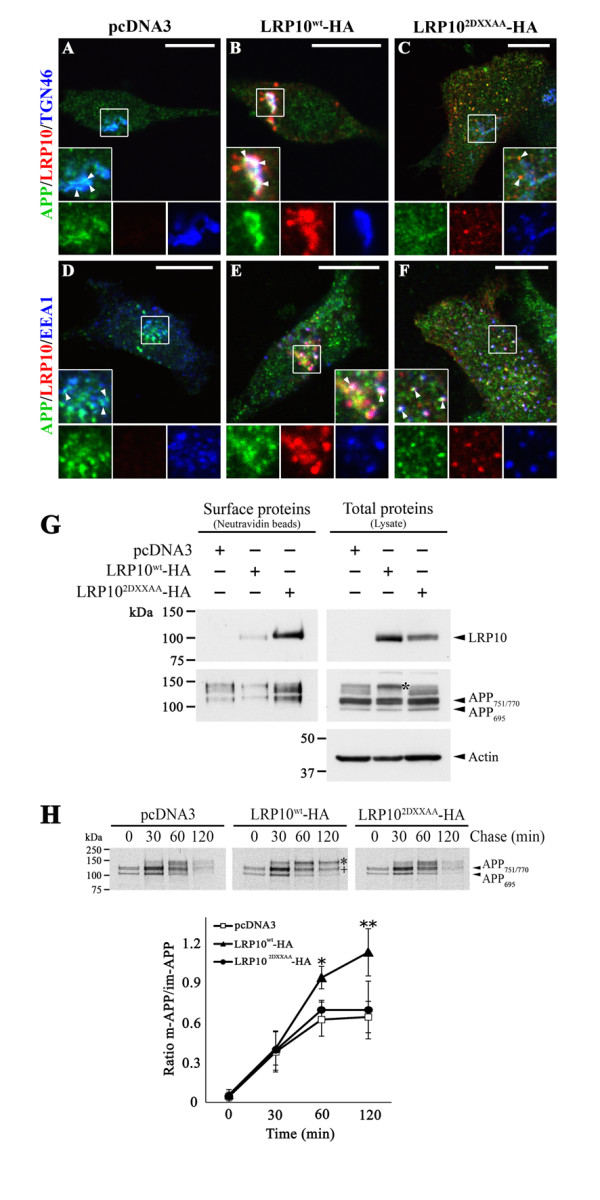
**LRP10 affect the trafficking of APP in neuronal SH-SY5Y cells. (A-F)** LRP10 colocalized and altered the distribution of endogenous APP. SH-SY5Y cells were stably transfected with empty pcDNA3 vector (A, D) or high levels of HA-tagged-LRP10^wt^ (B, E) or -LRP10^2DXXAA^ (C, F). Cells were fixed, permeabilized, and immunostained with anti-APP (green), anti-HA (red), and anti-TGN46 or anti-EEA1 (blue) antibodies. The labeled cells were examined by confocal fluorescence microscopy. (A, D) In control cells (pcDNA3), endogenous APP was detected mainly in the Golgi region where it partially overlapped with TGN46 (A, inset arrowheads) and surrounding vesicles where it partially overlapped with EEA1 (D, inset, arrowheads). (B, E) LRP10^wt^-HA was detected in the TGN (B, inset) and surrounding EEA1-labeled endosomes (E, inset). Endogenous APP was mainly detected in the Golgi region, where it partially overlapped with LRP10^wt^-HA (B, inset). The merged image shows a clear overlap between LRP10^wt^-HA and endogenous APP in the TGN (B, arrowheads, insets). (C, F) LRP10^2DXXAA^-HA was redistributed mainly to peripheral EEA1-labeled early endosomes (F, inset). Endogenous APP was also detected in peripheral EEA1-labeled endosomes (F, inset), where it colocalized with HA-tagged LRP10^2DXXAA^ (F, arrowheads, inset). Scale bar, 10 μm. **(G)** The level of cell surface APP was altered by the expression of LRP10. SH-SY5Y cells stably expressing pcDNA3 vector or high levels of HA-tagged-LRP10^wt^ or -LRP10^2DXXAA^ were surface-biotinylated with sulfo-NHS-SS-biotin. Biotinylated proteins were precipitated with neutravidin, and samples (Surface proteins) were analyzed by immunoblotting with antisera directed against HA or APP. The total cell lysate (Total proteins) was also analyzed to assess APP and LRP10 expression levels. The three APP variants (APP_695_, APP_751_, and APP_770_) were detected by the anti-APP antibodies. The asterisk indicates an accumulation of mature APP (glycosylated APP_751/770_) in the presence of LRP10^wt^. Actin was used as a loading control. **(H)** The maturation of APP was altered in SH-SY5Y cells expressing LRP10^wt^. SH-SY5Y control cells or cells stably expressing LRP10^wt^ or LRP10^2DXXAA^ were pulse-labeled with [^35^ S]methionine for 5 min and chased at 37 °C for the indicated time. Radiolabeled APP was immunoprecipitated from the cell extracts and was analyzed by SDS-PAGE and autoradiography. The three APP variants (APP_695_, APP_751_, and APP_770_) are indicated by arrowheadss. Accumulations of mature APP (*, indicates mature APP_751/770_; +, indicates mature APP_695_) were observed in cells expressing LRP10^wt^. Graph of the ratio of mature (m) APP versus immature (im) APP in the indicated SH-SY5Y stable clones as determined by densitometric scanning of pulse-chase experiments exemplified in the autoradiogram above. The results indicated that there is a delayed turnover of the mature, fully glycosylated APP variants when they are co-expressed with LRP10^wt^. Results are expressed as means ± SD (n = 3). *, p < 0.05; **, p < 0.005 (compared to control cells).

To confirm the role of LRP10 in the redistribution of APP in a system that more closely mimics physiological conditions, we investigated the effect of LRP10 overexpression on the distribution of APP in SH-SY5Y cells, a human neuroblastoma cell line that expresses endogenous APP but little endogenous LRP10 protein (data not shown). We generated SH-SY5Y cells that stably expressed pcDNA3 (control) as well as high levels of HA-tagged LRP10^wt^ or HA-tagged LRP10^2DXXAA^. In the control cells, endogenous APP was localized in the TGN and in surrounding EEA1-labeled vesicles (Figure 6A, D). In cells expressing LRP10^wt^, APP labeling was stronger in the TGN compartment, where it colocalized with LRP10^wt^ (Figure 6B, Merge). As with HeLa cells, the expression of LRP10^2DXXAA^ in SH-SY5Y cells led to a redistribution of APP from the TGN to peripheral early endosomes, where it colocalized with HA-tagged LRP10^2DXXAA^ (Figure 6F, Merge). In these cells, plasma membrane labeling of LRP10^2DXXAA^ and APP was less prominent. These observations suggested that LRP10 affects the intracellular distribution of APP in human neuronal SH-SY5Y cells.

We next examined the effect of LRP10 on the redistribution and surface expression of APP using a cell surface biotinylation assay. SH-SY5Y cells stably expressing pcDNA3, LRP10^wt^, or LRP10^2DXXAA^ were incubated with a membrane-impermeable biotin reagent at 4°C. A fraction of the total cell lysates and the biotinylated proteins were resolved by SDS-PAGE analysis, and LRP10 and APP expression was determined by Western blotting (Figure 6G). As previously reported, APP_695_, APP_751_, and APP_770_, three variants of endogenous APP, were detected in these cells [16,17]. As predicted by the immunofluorescence observations, overexpression of LRP10^2DXXAA^ resulted in the accumulation of APP together with LRP10^2DXXAA^ on the cell surface (Figure 6G). In contrast, while a small amount of LRP10^wt^ was detected on the plasma membrane of SH-SY5Y cells overexpressing LRP10^wt^, the amount of APP on the cell surface was lower than that of control (pcDNA3) cells (Figure 6G). The cell lysates revealed that the levels of the APP variants were similar in the different SH-SY5Y clones (Figure 6G). Interestingly, overexpression of LRP10^wt^ resulted in the accumulation of a higher molecular weight form of APP compared to the control and LRP10^2DXXAA^ cells (Figure 6G, asterisks). This higher molecular weight form of APP corresponds to the mature (glycosylated) form of APP_751/770_[17,18]. The accumulation of the mature APP species in the presence of LRP10^wt^ was confirmed by studying the time course of APP maturation using ^35^ S]methionine pulse-chase experiments (Figure 6H). The emergence of mature forms of APP followed similar kinetics in cells without LRP10 and with LRP10^wt^ or LRP10^2DXXAA^ (30-min chase), but the half-life of the mature APP protein was longer in the presence of LRP10^wt^ (Figure 6H), as shown by the ratio of signal intensities for mature versus immature APP species (120-min chase; p < 0.005) (Figure 6H). In summary, overexpression of LRP10^wt^ resulted in higher amounts of mature APP, with an accumulation of APP in the TGN and lower amounts on the cell surface, suggesting that APP is retained in the TGN. In contrast, overexpression of LRP10^2DXXAA^ resulted in higher amounts of APP on the cell surface and an accumulation in endosomes, suggesting that APP recycling to the TGN was impaired.

### LRP10 alters the processing of APP in SH-SY5Y cells

**Figure 7:**
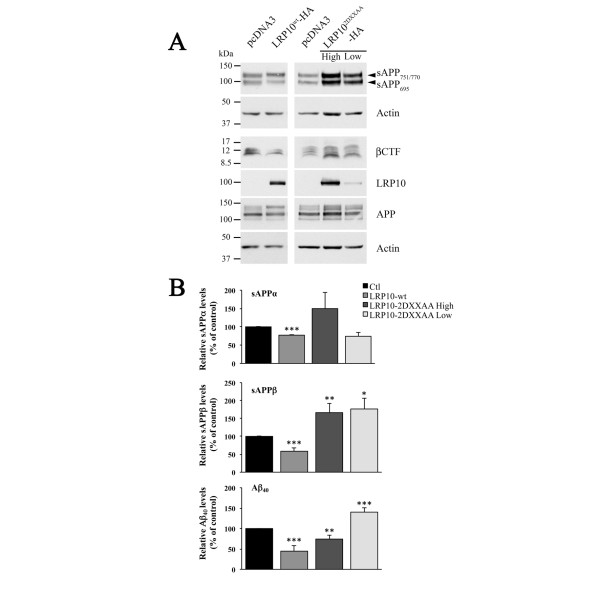
**LRP10 overexpression alters APP processing in SH-SY5Y cells.** APP cleavage products in SH-SY5Y stable clones expressing pcDNA3 vector alone (Ctl) or HA-tagged LRP10^wt^ or LRP10 trafficking mutant LRP10^2DXXAA^. **(A)** Representative Western blot of total sAPP secreted in the media and of β-CTF and total APP in the cell lysates of the indicated SH-SY5Y stable clones. Actin was used as loading control. **(B)** AlphaLISA quantitative analysis of sAPPα, sAPPβ, and Aβ_40_ in the media of the SH-SY5Y stable clones expressing pcDNA3 vector alone (Ctl), high level of HA-tagged LRP10^wt^ (LRP10^wt^) or low and high levels of LRP10 trafficking mutant (LRP10^2DXXAA^). Results are expressed as means ± SD (n = 3). *, p < 0.01; **, p < 0.005; ***, p < 0.001 (compared with control cells).

To establish a causal role for LRP10 in APP trafficking, we examined the effect of LRP10 overexpression on the processing of APP in SH-SY5Y cells stably expressing pcDNA3, HA-tagged LRP10^wt^, or HA-tagged LRP10^2DXXAA^. We investigated whether LRP10 altered the generation of APP processing products using Western blotting to detect total soluble APP fragment (sAPPα + sAPPβ) as well as intracellular β-carboxy-terminal fragment (β-CTF) levels (Figure 7A). In parallel, AlphaLISA assays were performed to specifically detect and quantify the secretion of sAPPα, sAPPβ, and the Aβ_40_ peptide into the cell culture media (Figure 7B). The expression of LRP10^wt^ in SH-SY5Y cells resulted in lower total sAPP and β-CTF levels as assessed by Western blotting (Figure 7A). This was confirmed and quantified by AlphaLISA (Figure 7B). Compared to control cells, LRP10^wt^ expression caused a 23% decrease (p < 0.001) in sAPPα, 42% (p < 0.001) in sAPPβ, and 55% (p < 0.001) in Aβ_40_ levels (Figure 7B). In contrast, the expression of LRP10^2DXXAA^ increased sAPP and β-CTF levels as assessed by Western blotting (Figure 7A). The AlphaLISA assays indicated that there was no significant effect on the secretion of sAPPα (p = 0.06) but that sAPPβ levels increased by 66% (p < 0.005) compared to control cells (Figure 7B). Unexpectedly, we observed a 26% decrease in Aβ_40_ levels (p < 0.005) (Figure 7B), suggesting lower γ-secretase cleavage of β-CTF. This was confirmed by the decrease in AICD fragments that arise as a co-product of β-CTF cleavage by γ-secretase (Additional file 3: Figure S3). To determine whether this effect was due to the high levels of LRP10^2DXXAA^ expression, we examined the APP processing products in SH-SY5Y cells stably expressing low levels of LRP10^2DXXAA^. As observed in SH-SY5Y stably expressing high levels of LRP10^2DXXAA^, Western blots revealed that there was an increase in sAPP and β-CTF levels (Figure 7A) while the AlphaLISA assays indicated that there was no significant effect on the secretion of sAPPα (p > 0.05) but an increase in sAPPβ (Figure 7B). However, unlike the SH-SY5Y clone expressing high levels of LRP10^2DXXAA^, Aβ_40_ levels increased by 40% (p < 0.001) compared to control cells, in SH-SY5Y cells stably expressing low levels of LRP10^2DXXAA^ (Figure 7B). We attempted to measure Aβ_42_ in the different SH-SY5Y clones, but the levels of endogenously produced Aβ_42_ were below reliable detection limits. In summary, the overexpression of LRP10^wt^ resulted in decreased non-amyloidogenic and amyloidogenic processing. The same results were observed in SH-SY5Y cells stably expressing low levels of LRP10^wt^ (data not shown). In contrast, low levels of LRP10^2DXXAA^ expression increased the amyloidogenic processing of APP. However, while high levels of LRP10^2DXXAA^ expression increased amyloidogenic processing, as indicated by the increases in sAPPβ and β-CTF, the Aβ_40_ levels was unexpectedly decreased. The expression of LRP10^2DXXAA^ thus increased the amyloidogenic processing of APP, which was expected since this LRP10 mutant increased the presence of APP in endosomes, the principal location of β-secretase. However, high LRP10^2DXXAA^ levels seemed to affect the γ-secretase cleavage of β-CTF and in turn to reduce Aβ_40_ production.

Given that the overexpression of LRP10 decreased APP processing, we theorized that a decrease in LRP10 expression would result in the opposite effect. To test this possibility, we performed siRNA experiments in which LRP10 siRNA was transiently transfected into SH-SY5Y cells stably expressing low levels of HA-tagged LRP10. The siRNA treatment resulted in significantly lower LRP10 protein levels in these cells than in the control cells (Additional file 4: Figure S4). We used Western blots and AlphaLISA assays to determine the levels of β-CTF and Aβ_40_, respectively (Additional file 4: Figure S4). β-CTF levels were higher in the LRP10 knockdown cells than in the control cells (Supplemental Figure 4A). Similarly, Aβ_40_ levels were 17% higher in LRP10-depleted cells than in the control cells (p = 0.001, Supplemental Figure 4B). These results indicated that reducing LRP10 levels causes a significant increase in amyloidogenic processing of APP. The low transfection efficiency of SH-SY5Y cells as well as the presence of other APP receptors in these cells could explain the weak effect of the LRP10 knockdown on the production of Aβ_40._ In summary, aberrant LRP10 trafficking by mutations in the DXXLL motif or the loss of LRP10 expression led to altered APP processing and increased Aβ_40_ production.

### LRP10 levels are reduced in Alzheimer’s disease brains

**Figure 8:**
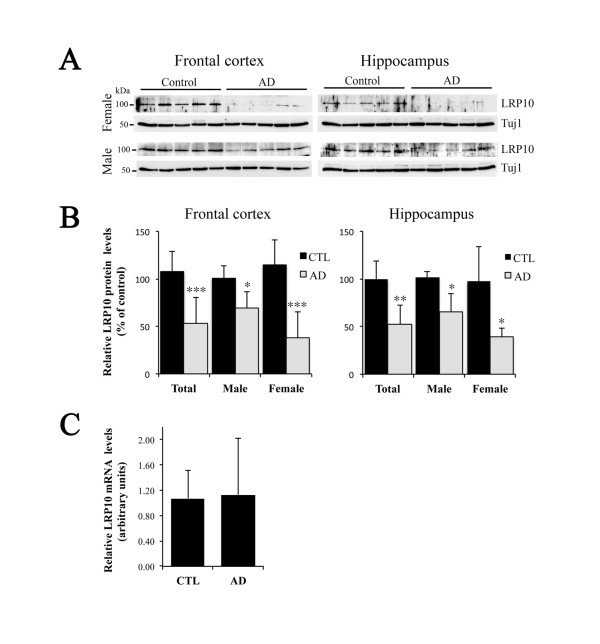
**LRP10 levels in healthy and AD brains.** Analysis of LRP10 protein and mRNA levels in healthy and AD brains. **(A)** LRP10 protein expression in the frontal cortex and hippocampus of healthy (Control) and Alzheimer’s disease (AD) patients was compared by Western blotting. Representative data showing lysates subjected to SDS-PAGE and immunoblotted with antisera directed against LRP10 and neuron-specific Class III β-tubulin (TUJ1). **(B)** Densitometric analysis of Western blots, such as those shown in (A)*,* normalized to the signal of TUJ1. Results are expressed as means ± SD (n ≥ 3). *, p < 0.01; **, p < 0.005; ***, p < 0.001 (compared with healthy patients). **(C)** LRP10 mRNA levels in the frontal cortex of healthy (CTL) and AD patients were compared by qRT-PCR. Total mRNA was reverse transcribed, and the levels of LRP10 cDNA were analyzed by qPCR with SYBR Green and were expressed relative to the endogenous control (RPL13) using the comparative CT method. Results are expressed as means ± SD (n = 5 samples, in duplicate). The difference between CTL and AD was not significant (p = 0.4).

Based on the results obtained with the SH-SY5Y and HeLa cells, LRP10 is a functional APP receptor that modulates APP trafficking and reduces its processing into Aβ. This finding raised the intriguing possibility that the increased Aβ production observed in patients with AD may involve the loss of LRP10 expression in the brain. This hypothesis was tested using post-mortem brain tissues from AD patients (Figure 8). Expression profiling of LRP10 in AD was performed by comparing LRP10 protein and mRNA levels in human frontal cortex and hippocampal tissue samples from ten autopsy-confirmed cases of AD and ten age-matched controls (Figure 8). The mean age at the time of death was 71.7 ± 7.2 years (range, 57–82 years) for the control subjects and 76.4 ± 3.1 years (range, 70–82 years) for the AD subjects. Patients suffering from AD expressed less LRP10 protein than the healthy controls as assessed by Western blotting (Figure 8A). Given that neuronal loss is a characteristic of AD brains, we normalized our Western blot findings to relevant markers to account for neuron-specific cell loss. When normalized to neuron-specific Class III β-tubulin (Tuj1), we found that LRP10 expression in the frontal cortex and hippocampus of AD brains was 50% and 47% lower (p < 0.001), respectively, than in control brains (Figure 8B). Interestingly, the decrease in LRP10 levels in AD brains from the female subjects was significantly higher than in those from the male subjects. LRP10 levels in the frontal cortex and hippocampus tissues of the male AD subjects were 31% and 36% lower than in the control subjects, but 67% and 60% lower in the female AD subjects (Figure 8B). To determine whether the decrease in LRP10 protein levels was due to a change in gene expression, total quantitative RT-PCR assays were performed on frontal cortex tissues from five AD and five control brains (Figure 8C). There was no significant difference between LRP10 mRNA levels in the AD samples and the control samples (normalized LRP10 expression: 1.07 ± 0.44 in AD brains vs. 1.13 ± 0.88 in control brains, p = 0.4). In summary, our results indicated that neuronal LRP10 protein levels are lower in the post-mortem brain tissues of AD patients.

## Discussion

LRP10 is a distinct member of the LDLR family that transits between the TGN, PM, and endosomes. However, the physiological role of LRP10 is unknown. The similarities between LRP10 and SorLA trafficking led us to hypothesize that LRP10 plays a role in APP metabolism. Our study showed that LRP10 is a novel APP sorting receptor that regulates APP trafficking and processing and provided evidence of its potential involvement in the pathophysiology of AD.

Our results revealed that there is a direct interaction between LRP10 and APP. Immunoprecipitation and *in vitro* pull-down assays indicated that the ectodomain of LRP10 is mainly involved in this interaction, much like SorLA and APoER2, two other APP receptors that interact with APP via their luminal domains [19,20]. Further studies will be needed to map the precise APP binding regions in LRP10. A confocal microscopy analysis indicated that exogenous APP in HeLa cells and endogenous APP in human neuronal SH-SY5Y cells mainly colocalizes with LRP10 in the TGN and, to a lesser extent, in early endosomes, suggesting that LRP10 and APP interact in these subcellular compartments.

Various LDLR members have been shown to be involved in the regulation of APP trafficking [2]. Our findings uncover LRP10 as a new LDLR member implicated in APP sorting. This is supported by the APP phenotypes resulting from the overexpression of wild-type LRP10 as well as the LRP10 trafficking mutant. The overexpression of LRP10^wt^ in neuronal SH-SY5Y cells resulted in an accumulation of APP in the TGN concomitant with a decrease at the cell surface as well as higher amounts of mature APP with a longer half-life. This suggested that the time of residence of APP in the TGN is prolonged by either the retention of APP molecules en route through the TGN to the cell surface or by the retrograde transport of internalized APP from the endosomes to the TGN. The second possibility is in line with a proposed role for LRP10 in endosome-to-Golgi trafficking [13,21]. However, there were no clear differences in the appearance of internalized APP in the TGN of HeLa cells transfected with APP in the absence or presence of LRP10^wt^, suggesting that LRP10 may control the exit of APP from the TGN. A retention of APP in the Golgi should cause an accumulation of mature APP molecules in the cell. This possibility was supported by the fact that SH-SY5Y cells stably expressing LRP10^wt^ displayed higher levels of mature APP molecules than control cells or cells expressing the LRP10^2DXXAA^ trafficking mutant. This finding implies that the exit of APP from the ER to the Golgi was normal but that the ability to transit to more distal compartments was blocked by LRP10^wt^ proteins, thus increasing the amount of fully glycosylated APP molecules in the cell. This possibility was further supported by the fact that LRP10^wt^ inhibits APP processing and, consequently, reduces the turnover rate of mature APP. Further studies are needed to clearly identify the trafficking steps regulated by LRP10 as well as the molecular mechanisms involved.

The ability of LRP10 to regulate APP routing was confirmed by targeting LRP10 to the endosomes, which caused an accumulation of APP in the same compartment. We showed that the expression of mutated LRP10^2DXXAA^, which could not bind GGA and AP1/AP2 proteins, impairs APP recycling to the TGN, since we observed that this LRP10 mutant is unable to retrogradely transport it out of the endosomes. Previous studies by the group of Kornfeld [15] using a chimera containing the ectodomain of LRP4 and the cytoplasmic tail of LRP9 (mouse LRP10) indicated that the mutation of the DXXLL motifs of LRP9 causes a decrease in its internalization rate in CHO cells [15], which could explain the accumulation of LRP10^2DXXAA^ and APP at the cell surface. Our IF uptake assays, which indicated that there is a delay in the internalization of FLAG-LRP10^2DXXAA^ compared to FLAG-LRP10^wt^ provided support for this possibility. As such, the enhanced expression of LRP10^2DXXAA^ on the cell surface would not be caused by mistargeting of LRP10 to the plasma membrane due to a disrupted AP1/2, GGA-binding motif, but to slower internalization from the cell surface. These findings suggested that APP is a likely physiological target for LRP10-mediated protein sorting in neurons.

Mounting evidence has shown that alterations in the intracellular distribution of APP have a direct impact on amyloidogenic and non-amyloidogenic processing [2]. APP targeting and time of residence in endosomes are known to modulate β-secretase cleavage and Aβ levels, while the transport of APP to the cell surface modulates its cleavage by α-secretase [2,22]. The relevance of LRP10-mediated Golgi retention of APP was confirmed by the distinct effect that LRP10^wt^ and LRP10^2DXXAA^ had on Aβ production. The retention processes induced by LRP10^wt^ would allow for more APP to be kept in circulation for longer times and to be kept away from endosomes or the PM, where it would be processed. The lower levels of amyloidogenic and non-amyloidogenic processing products detected in these cells corroborated this hypothesis. The relevance of LRP10-mediated Golgi retention/retrieval of APP was confirmed by the significant increase in amyloidogenic processing induced by siRNA depletion of LRP10. We also showed that the targeting and retention of APP in endosomes when LRP10^2DXXAA^ is expressed increases APP cleavage by the amyloidogenic pathway but does not significantly affect non-amyloidogenic processing. As such, APP cleavage by endosomal β-secretase increased and resulted in the production of more Aβ. LRP10 also affected β-CTF cleavage, with high expression levels of LRP10^2DXXAA^ appearing to increase sAPPβ and β-CTF levels but decrease Aβ and AICD levels, suggesting lower γ-secretase cleavage of β-CTF. Interestingly, previous studies have indicated that members of the LDLR family such as LRP and SorLA interact with and are cleaved by γ-secretase [23,24] and also compete with APP for this enzyme [23]. LRP10 may thus be either a substrate for γ-secretase and compete with APP for this enzyme or might affect γ-secretase distribution and activity. We intend to investigate these possibilities in future studies.

**Figure 9:**
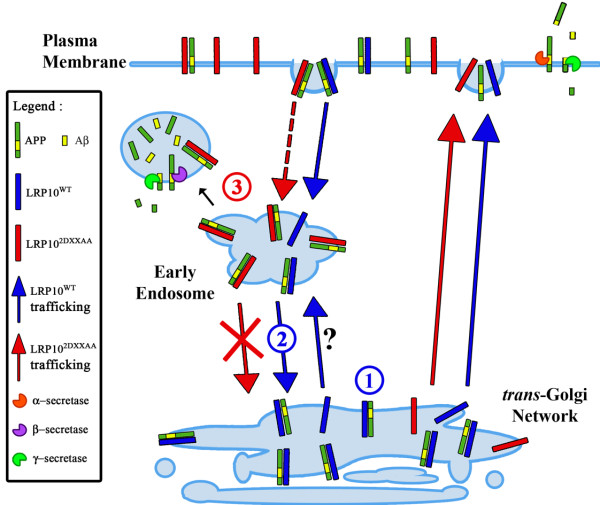
**Model for the role of LRP10 in APP trafficking.** LRP10 is transported from the Golgi to the plasma membrane in the secretory pathway independent of the DXXLL motifs. LRP10^wt^ is rapidly internalized from the cell surface into early endosomes where it is efficiently recycled back to the Golgi. LRP10 may also traffic directly from the TGN to the endosomes. LRP10^2DXXAA^, which is unable to associate with GGAs and AP1/2, is slowly internalized from the plasma membrane and accumulates in the early endosomes, since it is incapable of recycling to the TGN. APP is constitutively transported to the plasma membrane and internalized in endosomes. Interaction of APP with LRP10^wt^ prolonged its presence in the TGN. LRP10 could trap APP in the TGN (1), reducing the amount of APP transported to the cell surface for non-amyloidogenic processing by α- and γ-secretases. In addition, LRP10 may shuttle APP from early endosome back to the TGN (2), reducing its amyloidogenic processing by β- and γ-secretases. LRP10^2DXXAA^ traps APP in endosomes (3), resulting in enhanced accessibility to amyloidogenic secretases and thus processing into Aβ.

In summary, we propose a model whereby LRP10^wt^ is a sorting receptor that cycles between the TGN and the endosomes by transiting through the plasma membrane (Figure 9). LRP10^wt^ would traffic from the TGN to the plasma membrane via the constitutive secretory pathway. LRP10^wt^ would next internalize from the cell surface into early endosomes where it would recycle back to the TGN (Figure 9). However, we cannot exclude the possibility that LRP10^wt^ could also traffic from the TGN directly to endosomes and next be retrogradely transported to the TGN or anterogradely transported to the plasma membrane. Through its direct interaction with APP, LRP10^wt^ would prolong the time of residence of APP in the TGN either by TGN retrieval or retention and such, reduce the presence of APP at the cell surface and in endosomes as well as its cleavage by the secretases in these compartments (Figure 9). LRP10^2DXXAA^, which cannot be retrogradely transported from the endosomes to the Golgi, would accumulate with APP in the endosomes (Figure 9). This mislocalization would promote access to the amyloidogenic secretases in endosomes, leading to a higher production of Aβ (Figure 9). Therefore, changes in LRP10 distribution or expression would modify the balance between amyloidogenic and non-amyloidogenic processing.

Several LDL receptor family members play an important role in amyloidogenic processing. Most of these LDLR are localized at the PM and modulate APP endocytosis rates and cleavage in endosomes [2,3]. To date, only SorLA has been reported to regulate APP trafficking to the TGN. SorLA, like LRP10, retains APP in the Golgi compartment, increases the half-life of mature APP, and reduces APP processing by β-secretase in the early endosome, thus decreasing the production of Aβ [8,9]. LRP10 and SorLA are distinct LDLR members. Their only common domain is a ligand-binding repeat. However, they are homologous in terms of their trafficking pathways and regulation of APP trafficking and processing and are both present at lower levels in AD. These observations suggest that the regulation of every trafficking steps targeting APP to distinct subcellular compartments is shared by many sorting receptors, which highlights the importance of proper APP distribution.

The relevance of LRP10 for APP processing and its potential contribution to the pathogenesis of AD is supported by the observation that LRP10 is expressed at much lower levels in the brains of AD patients, which would result in higher Aβ levels in these patients. Interestingly, we observed that LRP10 levels in the brains of female AD patients are significantly lower than those of male AD patients, suggesting that LRP10 may affect AD through a female-specific mechanism. This observation requires further confirmation since it was based on a limited sample size (5 men and 5 women). Nevertheless, similar observations of AD sexual dimorphisms have been reported for two other members of the LDLR family that are known to be APP receptors and that share trafficking homology with LRP10. Indeed, there is a stronger genetic linkage to SorLA and SorCS1 (a homolog of SorLA) in women than in men in AD populations [25,26]. Higher Aβ levels have also been observed in the brains of female *Sorcs1* hypomorphic mice but not in the brains of male mice [27]. The higher prevalence of alterations to these APP receptors (including LRP10) in women is an important outcome since women account for 72% of all AD cases. However, the reasons for this are poorly understood*.*

In summary, our results suggested that altered LRP10 activity may be a potential risk factor for AD. The reason for reduced LRP10 expression in AD individuals remains to be determined. LRP10 mRNA levels do not change in AD. As such, the lower LRP10 protein levels seen in AD are not due to LRP10 gene transcription problems but rather to changes in protein translation or trafficking and stability of the receptor. The identification of the molecular events responsible for the proper localization and expression of this receptor is thus crucial. Interestingly, decreases in Calnuc and GGA proteins, which modulate LRP10 trafficking and levels [13,21], have been associated with AD [28-30]. Efforts should now be focused on identifying and studying human genetic variants of LRP10 to determine whether they are associated with AD in a sex-dependent fashion. The identification of functional causative variants influencing LRP10 expression and/or levels may help clarify their role in the pathogenesis of AD and may lead to possible future therapeutic strategies.

## Conclusions

Our experiments identified LRP10 as a novel APP sorting receptor that protects against amyloidogenic processing of APP and the accumulation of the Aβ peptide. Consequently, the reduced LRP10 receptor expression observed in the human brain may increase Aβ production and plaque formation and may be a risk factor in AD.

## Materials and methods

### Antibodies and reagents

Anti-HA mouse monoclonal antibodies (mAbs) and rabbit polyclonal antibodies (pAbs) were purchased from Covance (Berkley, CA, USA). Anti-GFP mouse mAbs and rabbit pAbs were from Clontech (Mountain View, CA, USA) and Molecular Probes (Eugene, OR, USA), respectively. Mouse anti-APPA4 clone 22 C11 mAbs (a.a. 66–81, N-term.) and rabbit anti-APP pAbs (C-term.) were from Millipore (Billerica, MA, USA) and Invitrogen (Carlsbad, CA, USA), respectively. Rabbit anti-LRP10 pAbs were from Abnova (Walnut, CA, USA). Mouse anti-Flag M2 mAbs and rabbit anti-Flag pAbs were purchased from Sigma (Oakville, ON, Canada). Mouse anti-actin AC-40 mAbs were from Sigma (Oakville, ON, Canada). Rabbit anti-EEA1 pAbs were from Thermo Scientific (Ottawa, ON, Canada) and were used for IB while goat anti-EEA1 was from Santa Cruz Biotechnology (Santa Cruz, CA, USA) and was used for IF. Sheep anti-TGN46 pAbs were from Novus Biologicals (Littleton, CO, USA).

### DNA constructs

Mammalian expression vector pCMV6-Entry encoding the human LRP10 was purchased from OriGene (Rockville, MD, USA). LRP10 was subcloned in the pcDNA3.1 vector, and PCR*-*based mutagenesis was used to insert an HA-tag at the C-terminus of LRP10 or a FLAG-tag after the signal peptide of LRP10. The LRP10 double DXXLL mutant construct was generated as described previously [13,15]. LRP10 fragments containing the N-terminus (residues 1–470) or C-terminus (residues 434–712) were amplified by PCR and subcloned in pcDNA3. Mammalian expression vectors GFP-APP_695_ and GST-APP_695_ C-term (residues 647–695) were kindly provided by Dr. Ritva Tikkanen (University Clinic of Frankfurt, Germany) [31]. The APP_695_ fragment containing the N-terminus (residues 20–612) was amplified by PCR and subcloned in pET41a (Novagen). All constructs were sequenced before being used.

### Cell cultures and transfections

HeLa and SH-SY5Y cells were purchased from ATCC (American Type Culture Collection, Manassas, VA, USA). HEK cells were kindly provided by Dr. Alexandra Newton (University of California, San Diego, CA, USA). HEK and HeLa cells were grown in Dulbecco’s modified Eagle’s high glucose medium (Invitrogen) containing 10% fetal bovine serum (FBS) (Hyclone Laboratories, Logan, UT, USA) and 1% penicillin and streptomycin. SH-SY5Y cells were grown in MEM:Ham’s F12 (1:1) (Invitrogen) containing 10% FBS, 2 mM sodium pyruvate (Invitrogen), and 1% penicillin and streptomycin. HeLa cells were transfected using Fugene6 transfection reagent (Roche Diagnostics, Indianapolis, IN, USA), and HEK cells were transfected with Lipofectamine 2000 transfection reagent (Invitrogen), both according to the manufacturers’ instructions. SH-SY5Y cells were stably transfected using Fugene HD (Roche Diagnostics) according to the manufacturer’s instructions, with expression constructs for pcDNA3.1 or LRP10 variants (in pcDNA3.1) and selected using 400 μg/ml of geneticin (G418; Invitrogen). For siRNA transfection of SH-SY5Y cells, 2X10^6^ cells were transfected with siRNA using Amaxa® Nucleofector® Cell Line Kit V with the Nucleofector® system and program A-023, as recommended by the manufacturer (Amaxa, Lonza, Walkerville, MD, USA). Scrambled RNA oligos (scramble II duplex) and LRP10 siRNA were purchased from Dharmacon Research.

### Immunofluorescence

Cells were seeded on coverslips. Twelve hours after transfection, the cells were fixed in 3% paraformaldehyde (PFA; Electron Microscopy Science, Hatfield, PA, USA) in 100 mM phosphate buffer (pH 7.4) for 30 min, permeabilized with 0.1% Triton X-100 for 10 min, blocked with 10% goat serum for 30 min, and incubated with primary antibodies for 1 h at RT, followed by Alexa Fluor 594- or 488-conjugated antibodies (Molecular Probes) for 1 h at RT. The specimens were visualized using an inverted confocal laser-scanning microscope (FV1000, Olympus, Tokyo, Japan) equipped with a PlanApo 60x/1.42 oil immersion objective (Olympus). Olympus Fluoview software version 1.6b was used to acquire and analyze the images. The images were further processed using Adobe Photoshop (Adobe Systems, San Jose, CA, USA).

### Antibody uptake assays

HeLa cells transiently expressing APP_695_ and pcDNA3, LRP10^wt^-HA, or LRP10^2DXXAA^-HA, or expressing LRP10^wt^ or LRP10^2DXXAA^ tagged with an extracellular FLAG epitope were grown on glass coverslips. The cells were washed twice on ice with ice-cold DMEM. They were then incubated for 60 min on ice in cold DMEM containing an antibody against the ectodomain of APP (α-APP 22C11, 12 μg/ml) or against FLAG (2 μg/ml). Cells were then washed, incubated at 37°C in complete medium for different periods of time, fixed with 3% paraformaldehyde, and processed for immunocytochemistry.

### Glutathione S-transferase pull-down assays

GST fusion proteins were expressed in *E. coli* BL21 and purified on glutathione-Sepharose 4B beads (Pharmacia, Piscataway, NJ, USA) according to the manufacturer’s instructions. ^35^ S-labeled *in vitro* translation products of pcDNA3.1-human LRP10 were prepared using the TNT T7 rabbit reticulocyte Quick Coupled Transcription/Translation system (Promega, San Luis Obispo, CA, USA) in the presence of [^35^ S]EasyTag EXPRESS labeling mix (73% met/22% cys; >1000 Ci/mmol, Perkin Elmer). For the pull-down assays, GST fusion proteins (10 μg) immobilized on glutathione-Sepharose 4B beads were incubated for 2 h at 4°C in the presence of *in vitro* translated products in 20 mM Tris–HCl buffer (pH 7.4) containing 150 mM NaCl, 3 mM EDTA, 0.1% NP-40, 1 mM DTT, and protease inhibitors. The beads were washed four times in lysis buffer and were boiled in Laemmli sample buffer. The bound proteins were separated by SDS-PAGE and were detected by autoradiography.

### Biotinylation assay

SH-SY5Y cell lines stably expressing either pcDNA3, LRP10^wt^, or LRP10^2DXXAA^ were washed with PBS and were treated with membrane-impermeable sulfo-*N* hydroxysuccinimidobiotin (Pierce) in PBS for 30 min at 4°C. The biotin labeling was quenched with 50 μl/ml of quenching solution before the cells were lysed in buffer (50 mM Tris buffer (pH 7.4), 150 mM NaCl, 2 mM EDTA, 1% Triton X-100, and protease inhibitors). The biotinylated cell surface proteins were precipitated with NeutrAvidin Agarose (Amersham Pharmacia Biosciences) and were analyzed by SDS-PAGE and immunoblotting.

### Co-immunoprecipitation

HEK cells were plated in 60-mm culture dishes and transfected with the various constructs. After 48 h, the cells were lysed in 50 mM Tris buffer (pH 7.4) containing 150 mM NaCl, 1% NP-40, and protease inhibitors for 1 h at 4°C and were then centrifuged at 13,000x*g* for 20 min. The cleared supernatants were incubated with primary antibodies (1 μg antibody per mg of proteins) overnight at 4°C and then with protein A-sepharose (GE Healthcare, Piscataway, NJ, USA) or protein G-Sepharose (Zymed, San Francisco, CA, USA) beads for 1 h. The beads were washed three times in lysis buffer and were boiled in Laemmli sample buffer. Bound immune complexes were analyzed by SDS-PAGE and immunoblotting.

### Immunoblotting

The protein samples were boiled in Laemmli loading buffer, separated on 8% or 10% SDS-PAGE gels, and transferred to 0.45 μm pore-size nitrocellulose membranes (Perkin Elmer, Woodbridge, ON, Canada). The membranes were blocked in Tris-buffered saline (20 mM Tris–HCl, pH 7.4, 150 mM NaCl) containing 0.1% Tween 20 and 5% nonfat dry milk and were incubated with primary antibodies for 1 h at RT and then with horseradish peroxidase-conjugated goat anti-rabbit or anti-mouse IgG (Bio-Rad, Richmond, ON, Canada) for 45 min at RT and enhanced chemiluminescence detection reagent (Pierce Chemical, Rockford, IL, USA).

### Pulse-chase labeling

For the pulse-chase experiments, the cells were incubated in cysteine- and methionine-free medium (Sigma) for 30 min prior to biolabeling using 150 μCi of L-[^35^ S]cysteine and L- [^35^ S]methionine/ml (EasyTag EXPRESS [^35^ S]-labeling mix (73% met/22% cys; >1000 Ci/mmol, Perkin Elmer)) for 5 min. The cells were then washed in ice-cold cysteine/methionine-free medium, chased for various time in complete medium, and lysed. Lysates were precipitated with 1 μg of rabbit anti-APP pAbs (C-term.) overnight at 4°C and were then incubated with protein A-sepharose beads for 1 h. The beads were washed three times in lysis buffer and prepared for standard SDS-PAGE and autoradiography. Films were scanned in grayscale at a resolution of 600 dpi, and the bands were quantified using Image-Pro Plus 6.0 (MediaCybernetics, Silver Spring, MD, USA)

### APP processing products

The amount of sAPP, carboxyl-terminal fragments (CTF), and Aβ_40_ products were determined by Western blotting and/or AlphaLISA detection. AlphaLISA kits were kindly provided by PerkinElmer (AL254 for sAPPα, AL255 for sAPPβ, and AL275 for Aβ_40_). To detect sAPP by Western blotting, 4x10^6^ SH-SY5Y cells were seeded on 60-mm dishes and cultured for 48 h in complete media. The culture media was then replaced by 1.5 ml of serum-free MEM:Ham’s F12 (1:1). After 24 h, the conditioned medium was harvested and centrifuged at 500xg for 5 min at 4°C. Fifty μl of supernatant was boiled with Laemmli loading buffer and directly loaded on an SDS-PAGE gel. Immunoblotting was performed as described above using 22C11 antibodies. The cells in each dish in which secreted APP was collected were lysed in 50 mM Tris buffer (pH 7.4) containing 150 mM NaCl, 2 mM EDTA, 1% Triton X-100 and protease inhibitors in order to determine and compare the amount of cells in each dish. Ten μl of each lysates were loaded on SDS-PAGE gels and immunoblotted for actin.

For the β-CTF Western blot analyses, 80–120 μg of cell lysate was separated on a 16.5% Tris-Tricine gel and transferred to a nitrocellulose membrane (0.20 μm pore size) (350 mA for 35 min). Immunoblotting was performed as described above using anti-APP C-term antibody. For the AlphaLISA assays, cells were seeded in 96-well plates at a density of 2x10^5^ cells per well in 100 μl of SH-SY5Y culture media. After a 48-h incubation, the supernatants were harvested and the analytes were quantified in triplicate using 5 μl of supernatant per assay with an EnVision Plate Reader (PerkinElmer). The counts were converted into pg/ml using standard curves. The cells were lysed in 100 mM NaOH to determine the total protein concentration using the BCA method (Pierce). The total protein concentrations in the lysates were used to normalize the concentrations of the analytes for the various samples and clones.

### Human brain tissues

Human hippocampal and frontal cortex samples from ten autopsy-confirmed cases of AD and ten age-matched control individuals were obtained from the Douglas Hospital Brain Bank in Montreal, Quebec, Canada. The mean age at death was 76.4 ± 3.1 years for the AD patients and 71.7 ± 7.2 years for the control group. The mean ages were not significantly different. The postmortem interval was 22.9 ± 13.5 h for the AD patients and 21.6 ± 8.3 h for the control group, which was not significantly different. The AD cases had a clinical diagnosis of probable AD, which was confirmed by a neuropathological evaluation. Control cases had a clinical diagnosis of nondemented elderly patients. Films were scanned in grayscale at a resolution of 600 dpi, and the bands were quantified using Image-Pro Plus 6.0 (MediaCybernetics, Silver Spring, MD, USA).

### Tissue protein extraction

Brain tissue samples (30 mg per sample) were homogenized on ice in RIPA lysis buffer solution containing protease inhibitors for 10 s using a Polytron homogenizer and were incubated on ice for 30 min. The homogenates were centrifuged at 13,000x*g* for 20 min. The protein concentrations in the extracts were estimated using the Bradford method (Bio-Rad), and 50 μg of protein aliquots were stored at −80°C in Laemmli sample buffer until used. The samples were separated on 10% SDS–PAGE gels, were transferred to nitrocellulose membranes (0.45 μm pore size) and were immunoblotted as described above.

### Tissue RNA isolation

Total RNA was isolated from human frontal cortex tissues (30–50 mg per sample) using the RNeasy Lipid Tissue Mini kit (Qiagen, Toronto, ON, Canada), according to the manufacturer’s instructions. Briefly, the tissue samples were disrupted in Qiazol lysis reagent and homogenized for 1 min on ice using a Polytron homogenizer until a completely homogeneous lysate was obtained. Chloroform was added, and the homogenate was separated into aqueous and organic phase by centrifugation. The upper aqueous phase was removed and ethanol was added to it. The sample was then applied to an RNeasy mini spin column, which was washed several times. In the final step, the RNA was eluted with 50 μl of RNAse-free water. The RNA concentration of the samples was estimated based on *A*260 measurements.

### Quantitative real-time RT-PCR

cDNA was synthesized using 1 μg of DNase 1 (Invitrogen)-treated RNA and 0.5 μg of oligo(dt)12-18 primer (Invitrogen) and 200 U of sup II Reverse Transcriptase (Invitrogen) according to manufacturer’s instructions. A total of 25 μl of real-time RT-PCR reactions (2 μl of cDNA, 3.5 μl of 10 μM forward and reverse primers) were performed using PerfeCTa SYBR Green SuperMix with Low Rox (QUANTA Bioscience, Gaithersburg, MD, USA) and a Stratagene Mx3005P QPCR System. Primers were designed to selectively amplify human LRP10 and Ribosomal Protein L13 (RPL13) mRNA sequences and were selected according to the manufacturer’s guidelines. RPL13 is a good housekeeper for qRT-PCR studies in autopsy brain tissue samples from control and AD cases [32]. PCR primers were synthesized and purified by IDT (Coralville, IA, USA). The sequences of the PCR forward and reverse primers were as follows for human LRP10 and RPL13, respectively: 5'- GGGTAGACCACAGAAGCTCCGGG-3' (sense), 5'-GGGTTAAGCGCTCTGAGCCACAG-3' (antisense) and 5'-CTCGGCCCCCAAGAAGGGAGAC-3' (sense), 5'- CCATCCCAGGCCCAGTTGTTCC-3' (antisense). Samples from five healthy and five AD patients were analyzed in duplicate. The final mRNA levels of the genes being studied were normalized to *RPL13* expression using the comparative CT method (Stratagene) [33]. Results are expressed as the means ± SD of five independent controls and five AD brain samples analyzed in duplicate for each gene.

### Statistical analysis

Experiments were performed in triplicate and results are expressed as means ± SD. The statistical significance of differences between samples was assessed using the Student *t*-test. A p < 0.01 was considered significant.

## Competing interests

The authors do not have any competing interest to disclose.

## Authors' contributions

JB established the LRP10 stable cell lines, carried out the LRP10 knockdown, performed immunoprecipitation, immunofluorescence and qRT-PCR assays as well as the analysis of brain extract and APP processing products and help to draft the manuscript. CT performed the in vitro pull-down, pulse-chase and APP uptake immunofluorescence assays. MLB helped with the immunoprecipitation assays. AM and SD provided the AlphaLISA kits and valuable technical assistance to set up the AlphaLISA quantification assays for APP processing products. CL conceived the study, designed experiments, coordinated data analysis and prepared the manuscript. All authors read and approved the final manuscript.

## Supplementary Material

Additional file 1 ** Figure S1. Interaction of untagged APP with LRP10-HA wild-type and trafficking mutant.** Lysates from HEK cells transfected with untagged APP_695_ and HA-pcDNA3, HA-tagged LRP10^wt^ or LRP10^2DXXAA^ were immunoprecipitated with anti-HA antibody and immunoblotted with anti-APP antibody to detect LRP10 and APP, respectively. Click here for file

Additional file 2 ** Figure S2. Interaction of APP and LRP10 expressed separately and subsequently combined.** HEK cells transfected separately with either GFP or GFP-APP_695_ or LRP10-HA were lysed. Cell lysates were subsequently mixed followed by immunoprecipitation with anti-HA antibody using the same conditions as described in Figure 1. Immunoblots with polyclonal anti-HA and anti-GFP antibodies showed a weak post-lysis interaction between LRP10-HA and GFP-APP which indicated that the proteins interact *in vitro*. Click here for file

Additional file 3 ** Figure S3. Lower levels of AICD fragments were detected in SH-SY5Y cells expressing high levels of LRP10**^**2DXXAA**^**.** Western blot analysis of AICD, a co-product of β-CTF cleavage by γ-secretase, in the cell lysates of SH-SY5Y stable clones expressing pcDNA3 vector alone (Ctl) or high levels of HA-tagged trafficking mutant LRP10^2DXXAA^**.** Actin was used as a loading control. Click here for file

Additional file 4 ** Figure S4. LRP10 knockdown increases amyloidogenic cleavage.** LRP10-depleted cells contained higher levels of Aβ_40_ and β-CTF. SH-SY5Y stable clones expressing low levels of HA-tagged LRP10^wt^ were transfected with control (siCTL) or LRP10 siRNA (siLRP10) for 3 days. (A) Representative Western blots of β-CTF and LRP10 in cell lysates of the SH-SY5Y stable clones treated with control or LRP10 siRNA. Actin served as a loading control. (B) AlphaLISA quantitative analysis of Aβ_40_ in the media of the LRP10 low expressor SH-SY5Y stable clones transfected with control (siCTL) or LRP10 siRNA (siLRP10). Results are expressed as means ± SD (n = 3). ***, p < 0.001 (compared with control cells). Click here for file
