# Supplementary figures and images for: LDLR-related protein 10 (LRP10) regulates amyloid precursor protein (APP) trafficking and processing: evidence for a role in Alzheimer’s disease
Source: Mol Neurodegener. 2012 Jun 26;7:31. doi: 10.1186/1750-1326-7-31 (PMC3520120; doi:10.1186/1750-1326-7-31)

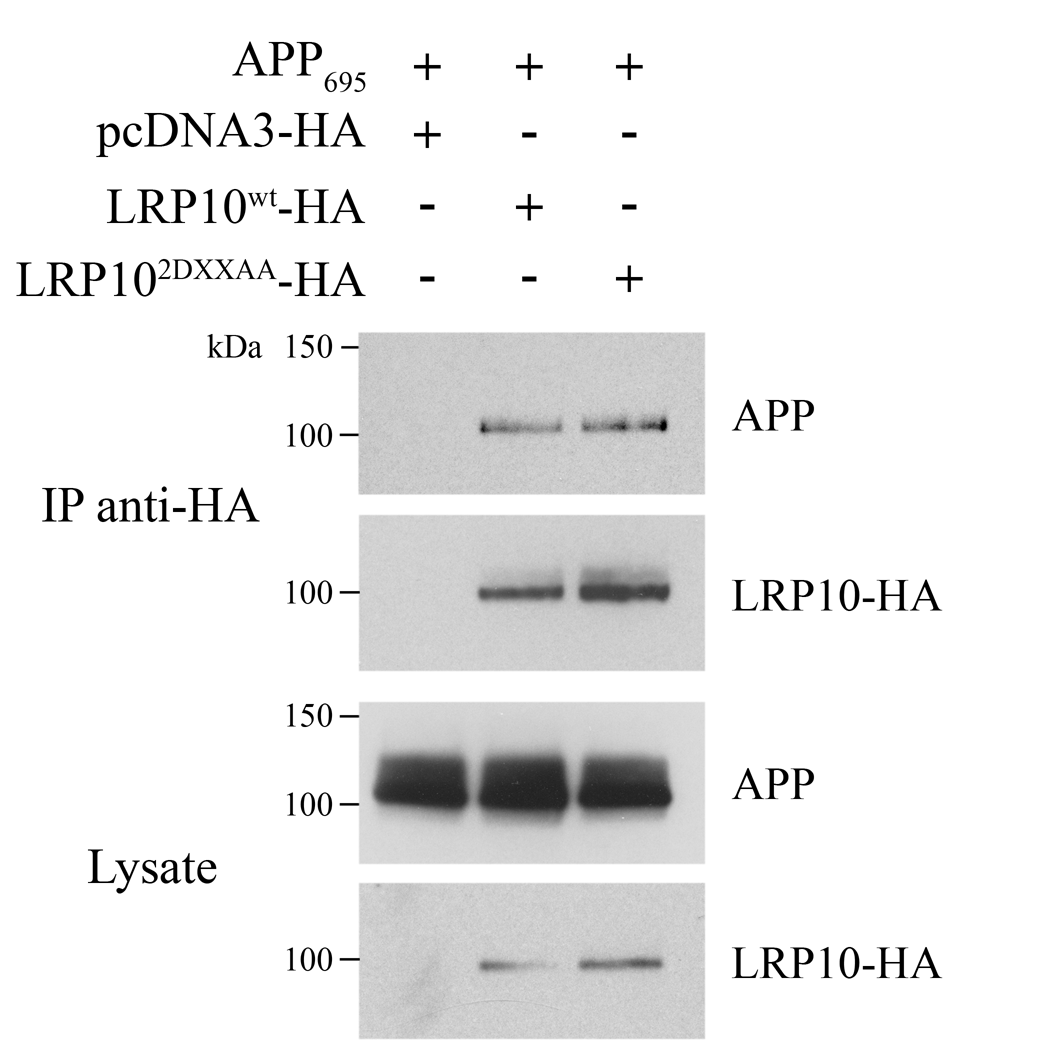

Supplement: Additional file 1 — Figure S1. Interaction of untagged APP with LRP10-HA wild-type and trafficking mutant. Lysates from HEK cells transfected with untagged APP695 and HA-pcDNA3, HA-tagged LRP10wt or LRP102DXXAA were immunoprecipitated with anti-HA antibody and immunoblotted with anti-APP antibody to detect LRP10 and APP, respectively. [file 1750-1326-7-31-S1.tiff]

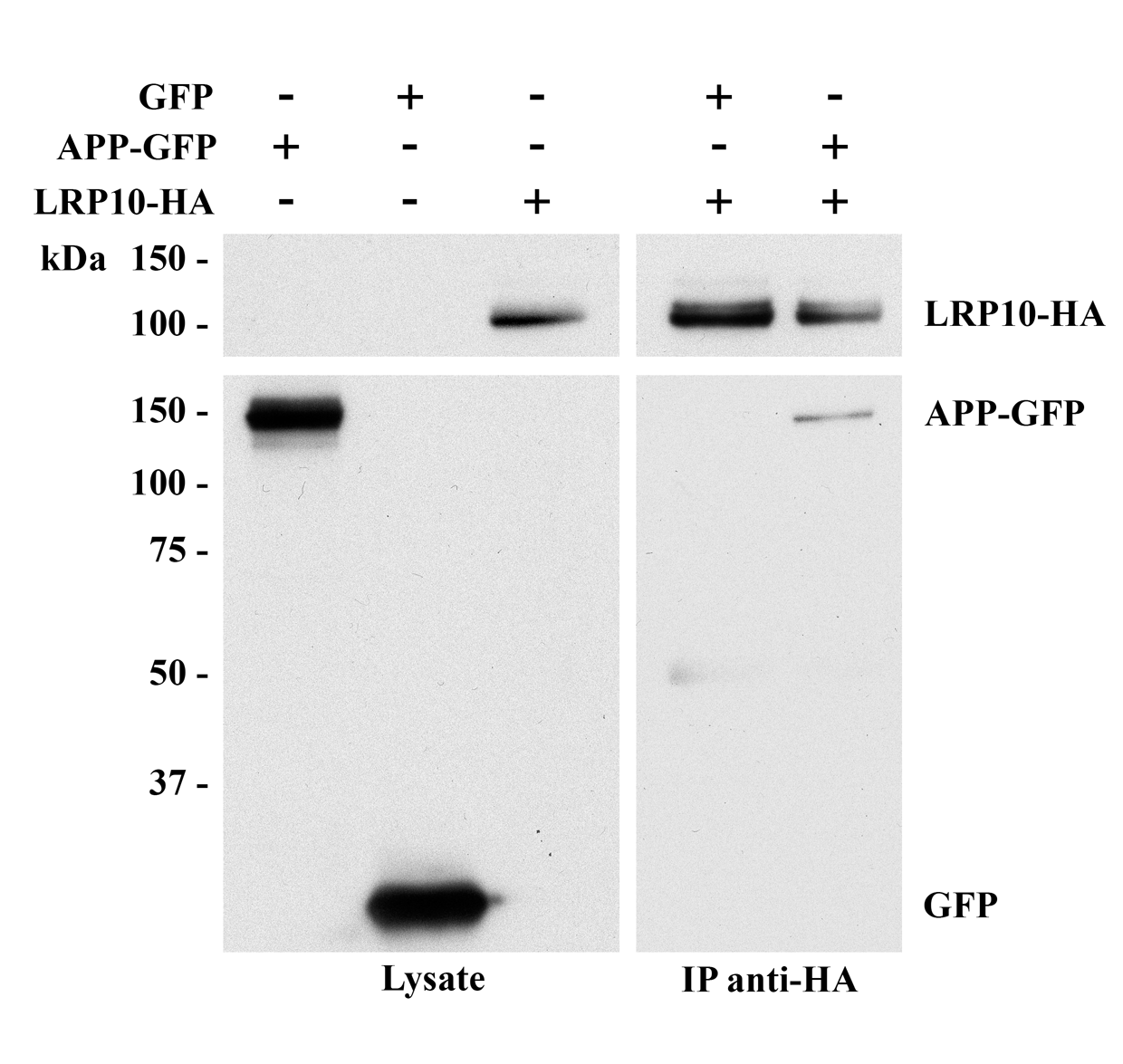

Supplement: Additional file 2 — Figure S2. Interaction of APP and LRP10 expressed separately and subsequently combined. HEK cells transfected separately with either GFP or GFP-APP695 or LRP10-HA were lysed. Cell lysates were subsequently mixed followed by immunoprecipitation with anti-HA antibody using the same conditions as described in Figure 1. Immunoblots with polyclonal anti-HA and anti-GFP antibodies showed a weak post-lysis interaction between LRP10-HA and GFP-APP which indicated that the proteins interact in vitro. [file 1750-1326-7-31-S2.tiff]

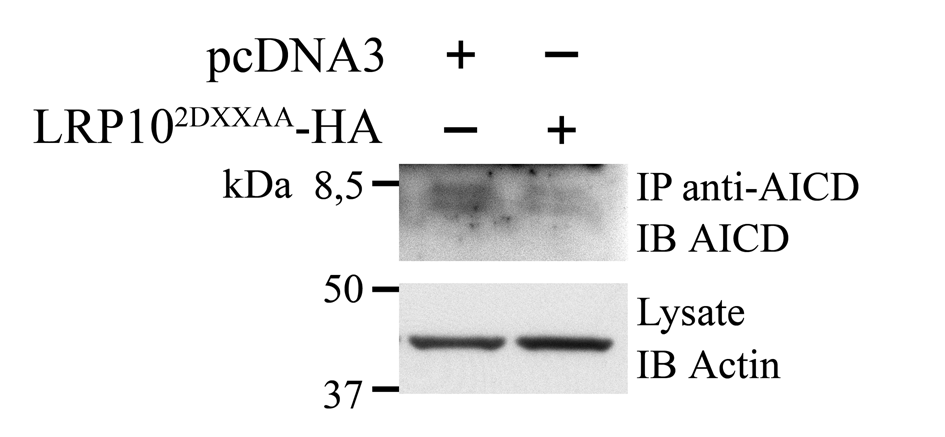

Supplement: Additional file 3 — Figure S3. Lower levels of AICD fragments were detected in SH-SY5Y cells expressing high levels of LRP102DXXAA. Western blot analysis of AICD, a co-product of β-CTF cleavage by γ-secretase, in the cell lysates of SH-SY5Y stable clones expressing pcDNA3 vector alone (Ctl) or high levels of HA-tagged trafficking mutant LRP102DXXAA. Actin was used as a loading control. [file 1750-1326-7-31-S3.tiff]

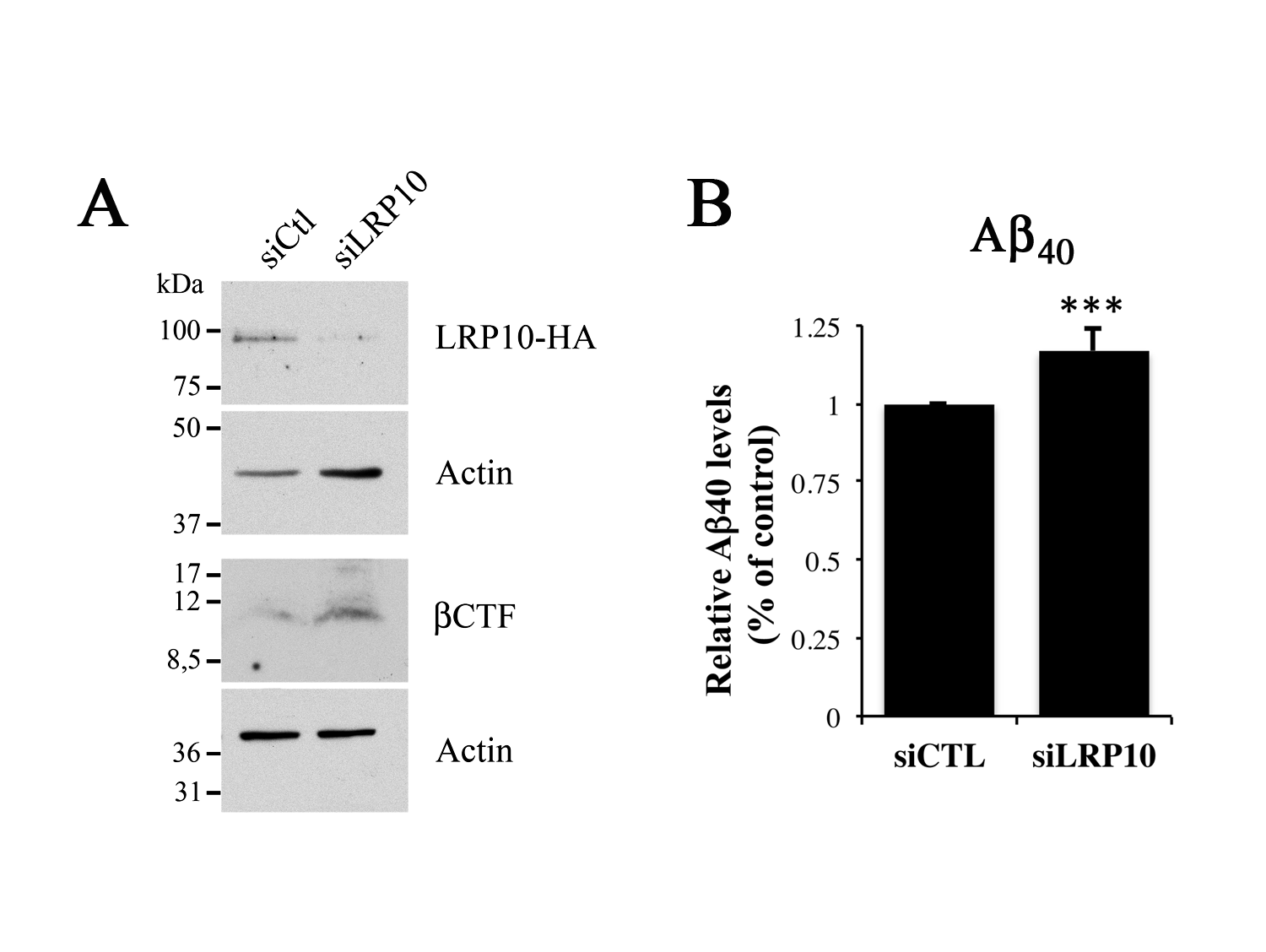

Supplement: Additional file 4 — Figure S4. LRP10 knockdown increases amyloidogenic cleavage. LRP10-depleted cells contained higher levels of Aβ40 and β-CTF. SH-SY5Y stable clones expressing low levels of HA-tagged LRP10wt were transfected with control (siCTL) or LRP10 siRNA (siLRP10) for 3 days. (A) Representative Western blots of β-CTF and LRP10 in cell lysates of the SH-SY5Y stable clones treated with control or LRP10 siRNA. Actin served as a loading control. (B) AlphaLISA quantitative analysis of Aβ40 in the media of the LRP10 low expressor SH-SY5Y stable clones transfected with control (siCTL) or LRP10 siRNA (siLRP10). Results are expressed as means ± SD (n = 3). ***, p < 0.001 (compared with control cells). [file 1750-1326-7-31-S4.tiff]
